# Adjuvant gemcitabine and concurrent radiation for patients with resected pancreatic cancer: a phase II study

**DOI:** 10.1038/sj.bjc.6603270

**Published:** 2006-07-25

**Authors:** A W Blackstock, F Mornex, C Partensky, L Descos, L D Case, S A Melin, E A Levine, G Mishra, S A Limentani, L A Kachnic, J E Tepper

**Affiliations:** 1 Department of Radiation Oncology, Wake Forest University School of Medicine, Medical Center Blvd, Winston-Salem, NC 27157, USA; 2 Centre Hospitalier Lyon Sud Radiation Oncology Department, Pierre Benite 69310, Lyon, France; 3 Blumenthal Cancer Center, 1000 Blythe Blvd #601, Charlotte, NC 28203, USA; 4 Boston University Medical Center, 88 East Newton St, EB 11, Boston, MA 02118, USA; 5 University of North Carolina, Manning Drive, Chapel Hill, NC 27599, USA

**Keywords:** pancreatic, adjuvant, gemcitabine, chemoradiation

## Abstract

The safety and efficacy of gemcitabine and concurrent radiation to the upper abdomen followed by weekly gemcitabine in patients with resected pancreatic cancer was determined. Patients with resected adenocarcinoma of the pancreas were treated with intravenous gemcitabine administered twice-weekly (40 mg m^−2^) for 5 weeks concurrent with upper abdominal radiation (50.4 Gy in 5½ weeks). At the completion of the chemoradiation, patients without disease progression were given gemcitabine (1000 mg m^−2^) weekly for two cycles. Each cycle consisted of 3 weeks of treatment followed by 1 week without treatment. Forty-seven patients were entered, 46 of whom are included in this analysis. Characteristics: median age 61 years (range 35–79); 24 females (58%); 73% stage T3/T4; and 70% lymph node positive. Grade III/IV gastrointestinal or haematologic toxicities were infrequent. The median survival was 18.3 months, while the median time to disease recurrence was 10.3 months. Twenty-four percent of patients were alive at 3 years. Only six of 34 patients with progression experienced local regional relapse as a component of the first site of failure. These results confirm the feasibility of delivering adjuvant concurrent gemcitabine and radiation to the upper abdomen. This strategy produced good local regional tumour control.

Cure with surgery alone for patients with operable pancreatic adenocarcinoma remains infrequent. A small randomised trial reported by the Gastrointestinal Study Group (GITSG) demonstrated an improvement in median and overall survival for patients treated with a surgical resection who were randomised to adjuvant 5-fluorouracil-based chemoradiation (*vs* observation) (1987). The chemoradiation consisted of bolus 5-fluorouracil (5-FU) and split course radiation to a total dose of 40 Gy. The median and 2-year survival for the surgery alone patients was 11 months and 18% *vs* 20 months and 43%, respectively, for the patients receiving adjuvant therapy. The European Organization for the Research and Treatment of Cancer (EORTC) attempted to replicate the results of the GITSG trial but failed to confirm a statistical benefit, despite a 4.5-month improvement in median survival for patients receiving adjuvant chemoradiation (Klinkenbijl *et al*, 1999). In an update of the European Study for Pancreatic Cancer (ESPAC 1) trial, six cycles of adjuvant 5-FU chemotherapy given alone or following split-course 5-FU-based chemoradiation appeared to provide a survival benefit over surgery alone (Neoptolemos *et al*, 2004). While the results of the ESPAC trial leave in question the role of radiation therapy in this setting, the use of chemoradiation reflects an accepted standard in the United States.

Gemcitabine is active as a single agent in the treatment of pancreatic cancer (Casper *et al*, 1994; Carmichael *et al*, 1996; Burris *et al*, 1997) with established radiation sensitising properties (Shewach and Lawrence, 1995, 1996; Lawrence *et al*, 1996, 1997, 1999; Joschko *et al*, 1997; Blackstock *et al*, 2001). While the vast majority of the reported phase I–III clinical trials have utilised gemcitabine as a single agent given weekly in a single dose, there are preclinical, pharmacologic and clinical data suggesting gemcitabine possesses equal, if not greater, cytotoxicity if given at a lower dose over a longer infusion time (Gandhi and Plunkett, 1990; Grunewald *et al*, 1991; Boven *et al*, 1993; Shewach *et al*, 1994).

Animal studies from the University of Michigan and MD Anderson Cancer Center suggest that maximum radiation sensitisation with gemcitabine is achieved with a lower dose administered twice-weekly (Mason *et al*, 1999; Fields *et al*, 2000). Fields *et al* treated squamous cell carcinoma VII xenografts with ionising radiation combined with isotoxic drug/radiation regimens (gemcitabine 800 mg kg^−1^ once weekly or 100 mg kg^−1^ twice-weekly). At 28 days post-treatment, tumours treated with twice-weekly gemcitabine and radiation were significantly smaller than tumours treated with the once weekly schedule (*P*<0.03). Related supporting clinical data come from Eisbruch *et al* (2001). Twenty-nine patients with unresectable head and neck cancer received a course of radiation concurrent with weekly infusions of low-dose gemcitabine. Tumour biopsies were obtained after the first gemcitabine infusion (before radiation started), and the intracellular concentrations of dFdCTP, the active metabolite of gemcitabine, were determined. Tumour dFdCTP levels and response rates were similar at all dose levels (50–300 mg m^−2^), demonstrating that significant tumour and normal tissue radiosensitisation can be achieved with ‘low-dose’ gemcitabine strategies.

Several phase I/II trials combining upper abdominal radiation and lower dose (40–90 mg m^−2^) twice-weekly gemcitabine have been reported, with each suggesting that this combination is safe and active (Blackstock *et al*, 1999; Pipas *et al*, 2001; Yavuz *et al*, 2001; Martenson *et al*, 2003). In a preoperative trial in pancreatic cancer from Joensuu *et al* (2004), the investigators observed a median survival of 25 months for patients taken to resection after receiving preoperative twice-weekly gemcitabine and radiation. While survival in the Cancer and Leukemia Group B (CALGB) trial of twice-weekly gemcitabine and radiation for locally advanced unresectable patients was only comparable to that expected with 5-FU-based chemoradiation, the regimen possessed moderate but acceptable toxicity and local control appeared improved (Blackstock *et al*, 2004).

This study was initiated to evaluate the combination of adjuvant radiation with concurrent twice-weekly gemcitabine followed by gemcitabine for patients with resected pancreatic cancer.

## PATIENTS AND METHODS

### Patient population

Patient's age ≥18 years with nonmetastatic surgically resected adenocarcinoma of the pancreas by pancreaticoduodenectomy (Whipple procedure or total pancreatectomy) were eligible. While patients with resected nodal disease were eligible, patients with focally positive (microscopically) margins (tumour at the margin) or patient's ≥8 weeks from surgery were ineligible. Inclusion criteria were the following: Eastern Cooperative Oncology Group performance status (PS) 0–2; adequate bone marrow – ANC (absolute neutrophil count) ≥1500 cells mm^−3^, platelet count ≥100 000 cells mm^−3^, and haemoglobin ≥10 g dl^−1^; kidney function (serum creatinine <2.0 mg dl^−1^) and liver function (serum total bilirubin <2 mg dl^−1^). Only patients able to maintain adequate oral nutrition and document a stable weight (no more than 5 lbs weight loss) for at least 2 weeks prior to enrollment were eligible. Patients with prior malignancy were ineligible for the study with the exception of those who had nonmelanoma skin cancer, *in situ* cancer of the cervix, or other cancer for which the patient had been disease free for ≥5 years.

All patients provided written informed consent according to federal and institutional guidelines. Institutional review board approvals were obtained at all participating sites.

### Treatment and evaluation

A complete course of therapy was defined as a total of 18 weeks or three cycles. Cycle 1 (weeks 1–6) incorporated the chemoradiation portion of the trial. Gemcitabine was delivered intravenously at a dose of 40 mg m^−2^ over 30 min, twice-weekly on either Monday and Thursday or Tuesday and Friday 30 min prior to the radiation therapy. During the concurrent chemoradiation, the gemcitabine was held for 1 week if the ANC was <1500 *μ*l^−1^ or the platelets were <100 000 mm^−3^ at the scheduled Monday dosing (the radiation continued). If the ANC or platelets had not recovered after the 1 week break, the gemcitabine and radiation were held until the ANC or platelets recovered and then restarted (no gemcitabine dose reductions). Gemcitabine doses held due to toxicity were not made up. Preoperative CT scans of the abdomen and surgical clips placed at the time of surgery were used to delineate treatment volumes. An initial 45 Gy was delivered to the tumour bed and peripancreatic nodal regions plus a 1.0–2.0 cm margin. The celiac axis was treated at the discretion of the radiation oncologist. The boost volume included the original tumour bed volume with a 2.0 cm margin. This volume received an additional 5.4 Gy. The specific design and configuration of the fields were individualised based upon the volume and location of the disease. Four field beam arrangements and 10–15 mV photon energies were required. In general, a four-field approach utilised anterior–posterior and left and right lateral beams. The spinal cord dose was limited to 45 Gy. In order to decrease hepatic irradiation, the anterior–posterior (AP/PA) fields were generally weighted more heavily than the laterals.

During cycles 2–3, gemcitabine was administered intravenously at a dose of 1000 mg m^−2^ over 30 min weekly for 3 weeks followed by 1 week of rest × 2 cycles. Within a cycle, if the ANC was between 500 and 999 *μ*l^−1^or the platelet count was between 50 000 and 99 999 mm^−3^ on the day of treatment, the dose of gemcitabine was reduced by 25%. The dose was held for an ANC less than 500 *μ*l^−1^ or a platelet count less than 50 000 mm^−3^. Patients with nonhaematologic toxicities grades 0–2 (and grade 3 nausea/vomiting) received full-dose gemcitabine. For grade 3 nonhaematologic toxicities other than nausea/vomiting, patients received either 75% of the gemcitabine dose or no treatment at the discretion of the treating physician. The gemcitabine dose was held for grade 4 nonhaematologic toxicities and missed doses were not made up.

Patients were evaluated for disease progression clinically and radiographically every 2 months the first year and then every 3 months. Weekly complete blood cell counts were required during treatment and the use of growth factors was discouraged. Disease progression was defined as the appearance of any new lesions on radiographic studies or in patients experiencing complications consistent with local–regional progression of disease, including new gastric outlet obstruction, duodenal obstruction, new bile duct obstruction, a decline in PS of at least one level, or the development of ascites not associated with gemcitabine therapy.

### Statistical considerations

The statistical analysis was performed at the Wake Forest University Comprehensive Cancer Center. The principal outcome measure used to quantify treatment effect was the Kaplan–Meier estimated 1-year survival. A two-stage phase II design was planned to test the null hypothesis that 1-year survival was ⩽35% *vs* the alternative hypothesis that 1-year survival was ≥50% with type I and II errors of 10%. The study was originally designed to accrue a maximum of 82 patients with an interim analysis after 53 patients. As the accrual was slower than expected, the study was closed with a sample size of 47 patients that provided an 78% power for testing the study hypothesis. The Kaplan–Meier method was also used to estimate the progression-free survival distribution. Overall survival was measured from the date of study entry until death due to any cause. Recurrence-free survival was measured from the date of study entry until disease progression or death from any cause. The log-rank test was used to assess differences in time-to-event distributions between patient subgroups.

Patient registration and data collection were managed by the Wake Forest University Statistical Center (US) and Centre Hospitalier Lyon Sud (France). Data quality was ensured by careful review of all data by the Wake Forest University Statistical Center, the Centre Hospitalier Lyon Sud staff and the study chairpersons.

## RESULTS

### Patient characteristics

Between June of 1999 and October of 2003, 47 patients with resected pancreatic cancer were entered onto this multicenter trial. This analysis incorporates data available from 46 patients, excluding one patient who was entered but was subsequently deemed ineligible (median follow-up 38.6 months). The pretreatment characteristics of the patients entered onto this trial are listed in Table 1. The median age was 61 years (range 35–79 years), all patients were resection margin negative and the median number of lymph nodes evaluated was 7 (range 1–27). The majority of patients (>70%) were lymph node positive and had advanced T-stage disease.

### Toxicity

Table 2 shows the grade 3 and 4 toxicities. Grade 3 and grade 4 haematologic toxicities, primarily neutropenia, occurred in 15 and 11% of patients, respectively. Grade 3 and 4 gastrointestinal toxicities, specifically nausea and vomiting, were uncommon, occurring in 13% and 0% of patients respectively. There was one death attributed to a gastrointestinal bleed that was scored as possibly treatment related. Forty-six percent of patients completed all 18 weeks of planned therapy while 74% of patients completed all planned chemoradiation and received ≥50% of the intended doses of maintenance chemotherapy.

### Survival and patterns of failure

**Figure 1:**
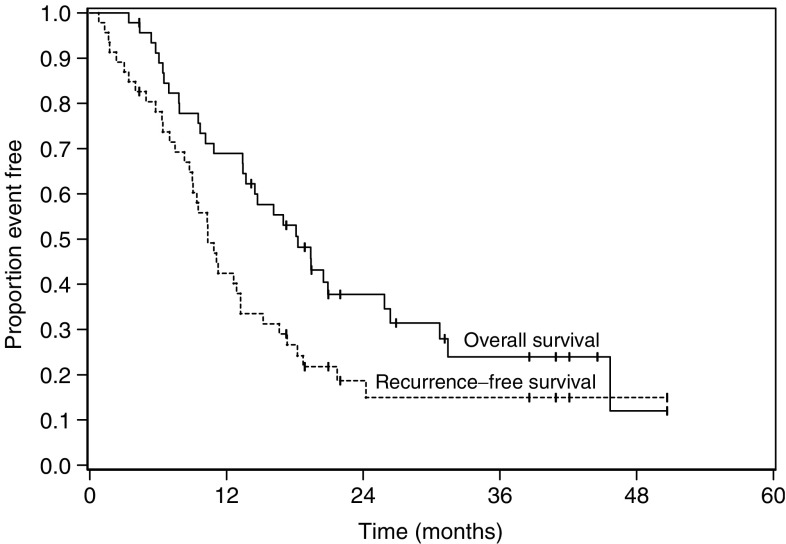
Overall and recurrence free survival.

The median survival and progression-free survival for the entire patient cohort were 18.3 (95% CI=13.7, 25.9) months and 10.3 (95% CI=9.0, 13.2) months, respectively. Sixty-nine percent (95% CI=0.55, 0.83) of patients were alive at 1 year and 24% were alive at 3 years (Figure 1). T-stage was prognostic; the time to recurrence for patients with T1–T2 disease was 16 months *vs* 9.2 months for patients with T3–T4 tumours (*P*=0.05). Overall median survival trended towards statistical significance as it related to T-stage, 30.7 months for the T1–T2 patients *vs* 14.6 for patients with T3–T4 tumours (*P*=0.06). Neither ethnicity nor gender was related to survival (Table 3); however, there were very few African-American patients in this study. While patients in the US experienced a slightly improved survival (nonstatistical), this likely relates to the overall more advanced T-stage and nodal stage disease found in patients enrolled in France.

At the time of this analysis, documented progression had occurred in 34 of the 46 patients. Three other patients died of their disease and were considered to have progressed. Distant progression represented the most frequent location of initial treatment failure (Table 4). Recurrence in the liver was a component of failure in 28 of 34 patients (82%) while six patients (18%) developed pulmonary metastasis. Ascites indicative of peritoneal carcinomatosis occurred in five of 34 (15%). Local–regional recurrence was observed in six of 34 patients who progressed (18%).

## DISCUSSION

The vast majority of the reports attempting to combine upper abdominal radiation and gemcitabine have been in the setting of locally advanced pancreatic cancer. In several instances, the reported toxicity has been significant. A phase II ECOG trial evaluating radiation and concurrent protracted venous infusion 5-FU (200 mg m^−2^ day^−1^) with weekly gemcitabine (50–100 mg m^−2^) was closed early due to excessive toxicity; three out of seven patients developed severe gastrointestinal toxicities (Talamonti *et al*, 2000). A phase II Cancer and Leukemia Group B trial evaluating a similar regimen found the toxicity to be acceptable (Mamon, personal communication). In data from MD Anderson Cancer Center, Wolff *et al* (2001) observed grade III/IV haematologic toxicity in over 50% of patients receiving weekly gemcitabine at doses ranging from 350 to 500 mg m^−2^, with 44% of the patients requiring hospital admission for severe nausea/vomiting and dehydration. An expanded retrospective review of patients receiving gemcitabine-based chemoradiation at MD Anderson, as reported by Crane *et al* (2001), further reflects the difficulties of combining the systemic toxicities of ‘full dose’ gemcitabine with the local–regional toxicities associated with radiation with the substantial radiation sensitisation of gemcitabine to the upper abdomen. The only trial to date able to achieve ‘full dose’ gemcitabine in this setting was reported by the University of Michigan utilising very specific radiation treatment guidelines (McGinn *et al*, 2001). As reported by McGinn *et al*, the treated tumour volume was restricted to the gross tumour with a 1.0 cm margin. No prophylactic nodal irradiation was given and the recommended phase II radiation dose was 36 Gy delivered in 2.4 Gy fractions.

The clinical experience with radiation and gemcitabine for pancreatic cancer in the adjuvant setting is more limited. Investigators at the University of Michigan found the strategy of fixing the dose of gemcitabine and escalating the ‘limited field’ radiation to be well tolerated in the adjuvant setting (Allen *et al*, 2004). In this phase I study of 32 patients with resected pancreatic cancer, gemcitabine at a dose of 1000 mg m^−2^ was given on days 1, 8 and 15 of a 28-day cycle concurrent with the radiation. The maximum tolerated dose of radiation was 39 Gy. While these are phase I data, the median survival was an acceptable 16.5 months. Van Laethem *et al*, in a feasibility study, found that gemcitabine (300 mg m^−2^) given weekly with split-course radiation (40 Gy/20 fractions over 6 weeks) was tolerable following three cycles of standard dose gemcitabine (1000 mg m^−2^ day 1 and 8 q 21 days) (Van Laethem *et al*, 2003). While limited to 22 patients, the median disease-free survival and overall survival was 6 and 15 months, respectively. In the present trial, 74% of patients were able to complete the entire chemoradiation portion of the study and receive ≥3 of the six planned doses of maintenance chemotherapy. This observation, along with the modest toxicity observed, suggests this regimen is well tolerated in the adjuvant setting.

In an attempt to put these results into perspective, Table 5 provides the results of selected phase III trials evaluating adjuvant chemoradiation strategies. The GITSG study found a benefit with the addition of adjuvant chemoradiation (*vs* observation) in a trial of 43 patients (1987). In a randomised study from the EORTC, which was not statistically significant, an almost 5-month survival advantage was observed for patients with pancreatic head cancers randomised to resection and adjuvant 5-FU-based chemoradiation *vs* observation (Klinkenbijl *et al*, 1999).

The ESPAC-1 trial attempted to evaluate several adjuvant strategies (Neoptolemos *et al*, 2004). Following resection, patients received either no therapy (observation), split-course radiation with 5-FU given during weeks 1 and 5 (no maintenance chemotherapy), six cycles of adjuvant/maintenance 5-FU chemotherapy, or split-course radiation with 5-FU followed by six cycles of adjuvant/maintenance 5-FU chemotherapy. The data from the ESPAC trial are difficult to interpret as the design of the trial allowed not only for randomisation between chemoradiation and observation but also a separate randomisation according to physician preference, for chemotherapy only, chemoradiation, or observation. Of the 541 patients, 346 (63%) had undergone either ‘background’ chemotherapy or chemoradiation prior to randomisation. In evaluating the most clinically relevant treatment arms, median survival for patients randomised to chemotherapy (alone) was 21.6 *vs* 19.9 months for those patients receiving chemoradiation followed by adjuvant/maintenance chemotherapy, suggesting no benefit with the addition of the radiation. Unfortunately, only 62% of the chemoradiation patients actually received the prescribed 40 Gy of radiation and, as stated by the authors, most of the protocol violations (50%) on the chemoradiation treatment arms were due to the patient's decision not to receive the assigned treatment.

In our study, the strategy of combining radiation with gemcitabine resulted in an acceptable median survival of over 18 months, despite the vast majority of patients having T3–T4 and/or node positive cancers. As shown in Table 5, the EORTC trial, which only allowed patients with T1–T2 tumours to be enrolled, achieved a median survival of 17 months in patients with pancreatic head cancers. While our trial only included 12 patients who would have been eligible for the EORTC study, the median survival for those patients was 31 months.

The results of our phase II trial may reflect an improved local–regional control of disease as well as an improved systemic approach. The 15% local–regional failure rate observed in the EORTC trial in both the observation and treatment arms is likely accurate for patients with node negative (53%) and/or T1–T2 periampullary/pancreatic head tumours. In our 46 patient trial, 18% of the patients with documented recurrences had local–regional failures, despite T3–T4 tumours and node positive patients accounting for 73 and 70% of the patients enrolled, respectively. The overall local recurrence (alone) rate in the ESPAC trial was 35% and likely reflects the anticipated local failure rate for patients with T3–T4 and/or node positive disease.

It is also possible that the two cycles of standard dose gemcitabine delivered following the chemoradiation may have improved outcomes. In a study of 368 patients with resected pancreatic cancer randomised to observation *vs* 6 months of adjuvant gemcitabine chemotherapy, Neuhaus *et al* (2005) observed a 14-month time to tumour recurrence for those patients receiving gemcitabine *vs* 7.5 months for those receiving no further therapy. The majority of patients in this trial had T3–T4 tumours and node positive disease. While survival results were not presented, these data suggest that gemcitabine may provide improved systemic disease control in the adjuvant setting. In the previously referenced CALGB experience utilising this strategy for patients with locally advanced unresectable disease, survival was not improved beyond that observed with 5-FU-based chemoradiation. However, this regimen in the adjuvant setting may be more promising. This perhaps reflects improvements in local–regional disease control with adjuvant gemcitabine-based chemoradiation along with improved systemic therapy (maintenance gemcitabine).

In conclusion, this phase II study confirms the feasibility and acceptable overall toxicity of adjuvant gemcitabine-based chemoradiation followed by maintenance gemcitabine chemotherapy for patients with resected pancreatic cancer. The time to disease recurrence and overall median survival were acceptable while local tumour control appeared improved.

## Figures and Tables

**Table 1:** Patient characteristics

**Characteristic**	**No.**	**(%)**
Total	46	(100)
		
Age – median (range)	61 (35–79)	
		
*Country*		
France	21	(46)
USA	25	(54)
		
*Performance status*		
ECOG 0	30	(65)
ECOG 1	15	(33)
ECOG 2	1	(2)
		
*Sex*		
Male	22	(48)
Female	24	(52)
		
*Ethnicity*		
Caucasian	43	(93)
African-American	3	(7)
		
*Tumour size*		
≥3.0 cm	27	(64)
<3.0 cm	15	(36)
Unknown	4	(—)
		
*Lymph node*		
Negative	13	(30)
Positive	30	(70)
Unknown	3	(—)
		
*Tumour stage*		
T1/T2	12	(27)
T3/T4	32	(73)
Unknown	2	(−)

**Table 2:** Grade III and IV toxicities experienced in 46 evaluable patients

	**Grade 3**		**Grade 4**	
**Toxicity**	***n***	**(%)**	***n***	**(%)**
*Haematologic*				
Neutrophils/granulocytes	7	(15)	5	(11)
Anaemia	8	(17)	2	(4)
Thrombocytopenia	6	(13)	1	(2)
				
*Constitutional*				
Fatigue (lethargy/malaise)	10	(22)	0	(0)
Weight loss	1	(2)	0	(0)
				
*Gastrointestinal*				
Anorexia	1	(2)	1	(2)
Nausea	6	(13)	0	(0)
Dehydration	0	(0)	0	(0)
Vomiting	2	(4)	0	(0)

**Table 3:** Overall survival (in months) by demographic characteristics

**Characteristic**	***n***	**Median**	**95% CI**	***P*-value**
Overall	46	18.3	13.7, 25.9	
				
*Age group*				0.44
<60	22	20.5	14.5, 31.4	
≥60	24	14.7	9.5, 25.9	
				
*Country*				0.21
France	21	17.0	7.8, 25.9	
USA	25	19.4	13.7, NA	
				
*Gender*				0.83
Female	24	19.4	10.9, 30.7	
Male	22	18.1	14.5, 25.9	
				
*Ethnicity*				0.10
African descent	3	13.7	4.4, 18.3	
Non African descent	43	19.4	13.4, 26.4	
				
*Performance status*				0.29
0	30	18.3	13.4, 25.9	
1–2	16	16.1	10.2, NA	
				
*Tumour size*				0.23
<3 cm	15	16.1	10.9, 20.5	
≥3 cm	27	19.4	13.7, 45.7	
				
*Nodal status*				0.15
Negative	13	30.7	13.7, NA	
Positive	30	15.4	9.7, 20.5	
				
*Stage*				0.07
T1,2	12	30.7	18.3, NA	
T3,4	32	14.6	10.2, 20.9	

NA, not applicable

**Table 4:** Sites of first recurrence

**Organ site**	**Number of patients**	**% of patients with documented progressions**
Liver	28	(82)
Pancreas/regional nodes	6	(18)
Ascites (carcinomatosis)	5	(15)
Pulmonary	3	(9)

A number of patients progressed synchronously in multiple sites.

**Table 5:** Phase III adjuvant chemoradiation trials for adenocarcinoma of the pancreatic head

**Trial**	**Treatment**	***n***	**% Stage ≥T3/%node (+)**	**Median survival (months)**
GITSG (1987)	Observation	22	35/28%	10.9
	40 Gy+5-FU^a^	21		20.0
				
EORTC (Klinkenbijl *et al*, 1999)	Observation	60	0/47%	12.6
	40 Gy+5-FU^b^	54		17.1
				
ESPAC (Neoptolemos *et al*, 2004)	Observation	69		16.9
	6 Cycles 5-FU	75	30/59%	21.6
	40 Gy+5-FU^b^	73		13.9
	40 Gy+5-FU^c^	72		19.9
				
^*^WFU/Lyon (phase II)	50 Gy+ Gemcitabine^d^	47	74/70%	18.3

a Patients received 5-FU (2 doses) during the radiation followed by 2 years of monthly therapy.

b Patients received 5-FU (2 doses) during the radiation only.

c Patients received 5-FU (2 doses) during the radiation followed by six additional months of 5-FU therapy.

d Patients received Gemcitabine (12 doses) during the radiation followed by two additional months of Gemcitabine.

5-FU, 5-fluorouracil.
